# Deputy Surgeon-General J. Liston Paul

**Published:** 1910-09-10

**Authors:** 


Deputy Surgeon-General J. Liston Paul, late of the Indian Army, a godson of Liston, died at Torquay last week. He was born at Elgin in 1827, and after studying at Edinburgh University and in Paris qualified as M.D., and entered the Medical Service of the Madras Army in 1851. After the usual regimental service he obtained the Professorship of Surgery in the Madras Medical College, which he filled for several years. He was also at this time engaged in a large practice in Madras. He retired comparatively early from the Indian Service with the honorary rank of Deputy Surgeon-General, though the substantive grade was offered to him by the Indian Government.

